# Activation of c-Jun N-Terminal Kinase (JNK) during Mitosis in Retinal Progenitor Cells

**DOI:** 10.1371/journal.pone.0034483

**Published:** 2012-04-04

**Authors:** Vinicius Toledo Ribas, Bruno Souza Gonçalves, Rafael Linden, Luciana Barreto Chiarini

**Affiliations:** Instituto de Biofísica Carlos Chagas Filho, UFRJ, Rio de Janeiro, Brasil; University of Washington, United States of America

## Abstract

Most studies of c-Jun N-terminal Kinase (JNK) activation in retinal tissue were done in the context of neurodegeneration. In this study, we investigated the behavior of JNK during mitosis of progenitor cells in the retina of newborn rats. Retinal explants from newborn rats were kept *in vitro* for 3 hours and under distinct treatments. Sections of retinal explants or freshly fixed retinal tissue were used to detect JNK phosphorylation by immunohistochemistry, and were examined through both fluorescence and confocal microscopy. Mitotic cells were identified by chromatin morphology, histone-H3 phosphorylation, and location in the retinal tissue. The subcellular localization of proteins was analyzed by double staining with both a DNA marker and an antibody to each protein. Phosphorylation of JNK was also examined by western blot. The results showed that in the retina of newborn rats (P1), JNK is phosphorylated during mitosis of progenitor cells, mainly during the early stages of mitosis. JNK1 and/or JNK2 were preferentially phosphorylated in mitotic cells. Inhibition of JNK induced cell cycle arrest, specifically in mitosis. Treatment with the JNK inhibitor decreased the number of cells in anaphase, but did not alter the number of cells in either prophase/prometaphase or metaphase. Moreover, cells with aberrant chromatin morphology were found after treatment with the JNK inhibitor. The data show, for the first time, that JNK is activated in mitotic progenitor cells of developing retinal tissue, suggesting a new role of JNK in the control of progenitor cell proliferation in the retina.

## Introduction

The retina is part of the central nervous system and is widely used as a model to study mechanisms of neurogenesis [1], due to knowledge of the spatio-temporal development of various retinal cell types. In newborn rats, the innermost (basal) cell layer is the ganglion cell layer (GCL) that contains only relatively differentiated ganglion cells [2], because displaced amacrine cells migrate postnatally into the GCL [3]. Further towards apical retina, there is an immature inner nuclear layer (INLi), followed by the proliferative neuroblastic layer (NBL). The NBL of neonatal rats contains both proliferating progenitor and postmitotic cells, including early developing horizontal cells [4]. The cell cycle in the proliferative zone of the retina, similar to other parts of the CNS, is tightly controlled and proceeds in synchrony with interkinetic migration of the progenitor cell nuclei along the depth of the NBL [5]. The phases of the cell cycle are easily identified in retinal progenitor cells, due to interkinetic nuclear migration [6]. DNA synthesis occurs in the inner side of the NBL, while mitosis is restricted to the outermost side of the NBL, as shown by immunohistochemical detection of phosphorylated histone-H3, which associates with condensing chromosomes of dividing cells [7]. The spatial segregation of the phases of the cell cycle along the interkinetic migration pathway facilitates experimental studies of cell proliferation in the retina. Nonetheless, the intracellular mechanisms that control phase transitions during the cell cycle are still poorly understood.

Mitogen-activated protein kinases (MAPK) are a group of enzymes that includes the extracellular-activated kinases (ERK), and the stress-activated protein kinases c-Jun N-terminal kinase (JNK) and p38. MAPK cascades are organized as a core signaling module consisting of three protein kinases: a MAP kinase kinase kinase (MKKK), a MAP kinase kinase (MKK), and a MAP kinase [8]. The JNK pathway is predominantly activated by stress stimuli such as cytokines, mitogens, osmotic stress and ultraviolet irradiation [9]–[12]. Ten JNK isoforms arise from alternative splicing of messenger RNA transcripts derived from three genes: *jnk1*, *jnk2* and *jnk3* [13]. JNK1 and JNK2 are ubiquitously expressed, while JNK3 is restricted to brain, heart and testis [10], [14]. Targeted gene disruption of each JNK has also disclosed differing functions of JNK1, JNK2 and JNK3 in distinct cell types, relative to gene expression, apoptosis, proliferation and other critical physiological responses [15]–[19]. While JNK1 and JNK2 are involved in the control of cell proliferation [20]–[24], JNK3 is important in the induction of neuronal cell death [15], [18], [19]. JNK is activated through phosphorylation of both residues Threonine-183 and Tyrosine-185, by the dual specificity enzymes MKK4 and MKK7 [25]. JNK can phosphorylate a variety of nuclear and cytoplasmic substrates. The major nuclear targets of JNK are transcription factors, in particular c-Jun, which is phosphorylated at both serine residues 63 and 73 [13], [26], [27]. In addition to nuclear targets, JNK can phosphorylate a variety of cytoplasmic substrates such as cytoskeletal proteins [28], MAP-2 [29] and proteins of the Bcl-2 family such as Bim [30].

Although classically described as a kinase responsive to cellular stress and implicated in induction of cell death, especially in the central nervous system [15], [18], [31], [32], several studies have demonstrated that the activity of JNK is also involved in cell survival and control of the cell cycle [16], [33], [34]. The data suggest that the JNK signaling pathway may be involved in controlling the M-phase of the cell cycle [35]–[40]. Most studies, however, were done in either tumor cell lines [20]–[22], [36]–[41] or dissociated cells [23], [24], [35], [42], and little is known of the role of this enzyme in cell proliferation in the central nervous system [43]–[45].

We use explants taken from retinal tissue of newborn rats to study mechanisms of cell proliferation in the developing central nervous system [4]. This preparation is advantageous because the retina of newborn rats differentiates *in vitro* in a similar rate as the retina *in situ*, maintains the structure of the tissue, contains cells at various stages of development, which can be easily recognized both by the position of their nuclei along the thickness of the retina and with immunohistochemical techniques and, finally, is easily amenable to pharmacological approaches [4], [46]. In the present study, we investigated the behavior of JNK during proliferation of progenitor cells in developing retinal tissue.

## Materials and Methods

All procedures complied with the Guidelines for the Care and Use of Laboratory Animals, from the ARVO Statement for the Use of Animals in Ophthalmic and Vision Research, and were approved by the Committee for the Use of Experimental Animals of the Institute of Biophysics (CAUAP/IBCCF/UFRJ # 046-09-2004).

### Materials

Dulbecco's Modified Eagle Medium (DMEM), Fetal Calf Serum (FCS) and gentamicin were from Gibco. Glutamine and horseradish peroxidase-conjugated anti-rabbit secondary antibody were from Sigma. SP600125 was from Tocris Bioscience. Antibodies to phospho-JNK (#9251) and phospho-histone-H3 (#9706) were from Cell Signaling Technology. Antibody to γ-tubulin (#ab11316) was from Abcam. Antibodies to phospho-JNK1/2 (#07-175) and JNK3 (#05-893) were from Upstate Biotechnology.

### Tissue Culture

Lister hooded rats at postnatal day 1 (P1), were killed painlessly by instantaneous decapitation and retinal explants were prepared as described previously [47], [48]. The neural retina was carefully dissected out of the eyeballs free of pigment epithelium with fine forceps. At least five fragments of about 1 mm^2^ (explants) were cut and placed in 25 ml tight-lidded Erlenmeyer flasks containing 5 ml DMEM and 5% FCS, 2 mM glutamine and 10 µg/ml gentamicin. Free-floating explants were cultured for 3 h at 37°C in a 5% CO2 atmosphere, in an orbital shaker at 70–80 rpm. Drugs (20 µM SP600125, 1 µM taxol) were added to the medium at the beginning of the incubation period. At the end of each experiment, the explants were fixed by immersion with 4% paraformaldehyde in phosphate buffer 0.1 M, pH 7.3, for at least 2 h, followed by cryoprotection in phosphate-buffered sucrose solutions (30%). The explants were oriented for transverse sections, and cut at 10 µm thickness in a cryostat.

For analysis *in situ* rats were killed by instantaneous decapitation, the eyeballs were removed and fixed immediately in 4% paraformaldehyde in 0.1 M phosphate buffer overnight, cryoprotected, and frozen sections were cut as above.

### Immunohistochemistry

Immunohistochemistry was done according to manufacturer's instructions. For antibody against phospho-histone-H3, antigen retrieval was carried out in citrate buffer pH 6. Sections of retinal explants were incubated with 0.5% Triton X- 100 in phosphate buffered-saline (PBS) pH 7.4, for 15 min, washed and incubated with 1% BSA in PBS for 30 min. Then, the sections were incubated overnight at 37°C with antibodies against phospho-JNK (1∶400), phospho-histone-H3 (1∶100), phospho-JNK1/2 (1∶100), JNK3 (1∶100) or γ-tubulin (1∶100). Then, the sections were incubated with the appropriate secondary antibodies for 1 h at 37°C (HRP-ABC kit - Vector) and developed with tyramide-Cy3. For double-labeling with phospho-histone-H3 or γ-tubulin, secondary antibody anti-mouse IgG conjugated with fluorochrome 488 was used (488 DyLigth – Jackson Immuno Research). The chromosomal DNA was stained with DAPI (1 µg/ml) or Topro-3 (1 µM; Invitrogen). For control of the phosphorylated epitope, some sections were treated with 10 U of calf intestine alkaline phosphatase in reaction buffer for 1 h at 37°C, prior to immunohistochemistry.

### Microscopy and cell counts

All images were made using an LSM510 confocal microscope coupled with an Zeiss Axiovert 200 M. Sections were also examined in a Zeiss Axiophot fluorescence microscope. Cells in M-phase were recognized by their location near the external limiting membrane of the retina, the morphology of the chromatin and also by the specific marker phospho-histone-H3. Counts were made at 1000× magnification, under oil immersion, in at least 3 distinct explants from each group, and data were pooled from at least 3 independent experiments. The percentage of cells stained for phospho-JNK was calculated among phospho-histone-H3 positive cells. For the analysis of phospho-JNK or phospho-histone-H3 along the mitotic stratum of the retina, labeled cells were counted and estimated per 100 µm of linear extent parallel to the retinal external surface [49]. For the analysis of phospho-JNK labeling in different mitotic stages, mitotic profiles were identified as either prophase/prometaphase, metaphase or anaphase, by the morphology of chromosomes stained with DAPI, then scored for phospho-JNK immunolabeling as either heavily or poorly labeled, or unlabeled. The percentages of either prophases/prometaphases, metaphases or anaphases were calculated among total mitotic profiles. In all experiments, at least 250 profiles were scored from each experimental group from at least three experiments [49]. Statistical analyses were done using one-way ANOVA, followed by Bonferroni's post-test to compare mean values between pairs of groups, or Student's *t*-test, as appropriate.

### Western Blotting

Total protein extracted from the retinal explants were separated by 12.5% SDS-PAGE, transferred onto nitrocellulose membrane, and probed with the same antibodies to phospho-JNK and phospho-histone-H3 used for immunohistochemistry, followed by a horseradish peroxidase-conjugated secondary antibody, and visualized using ECL-plus detection reagent (Amersham). The membranes were reblotted with a polyclonal antibody to β-actin (Abcam), as a loading control.

## Results

### JNK is phosphorylated during mitosis of retinal progenitor cells

**Figure 1:**
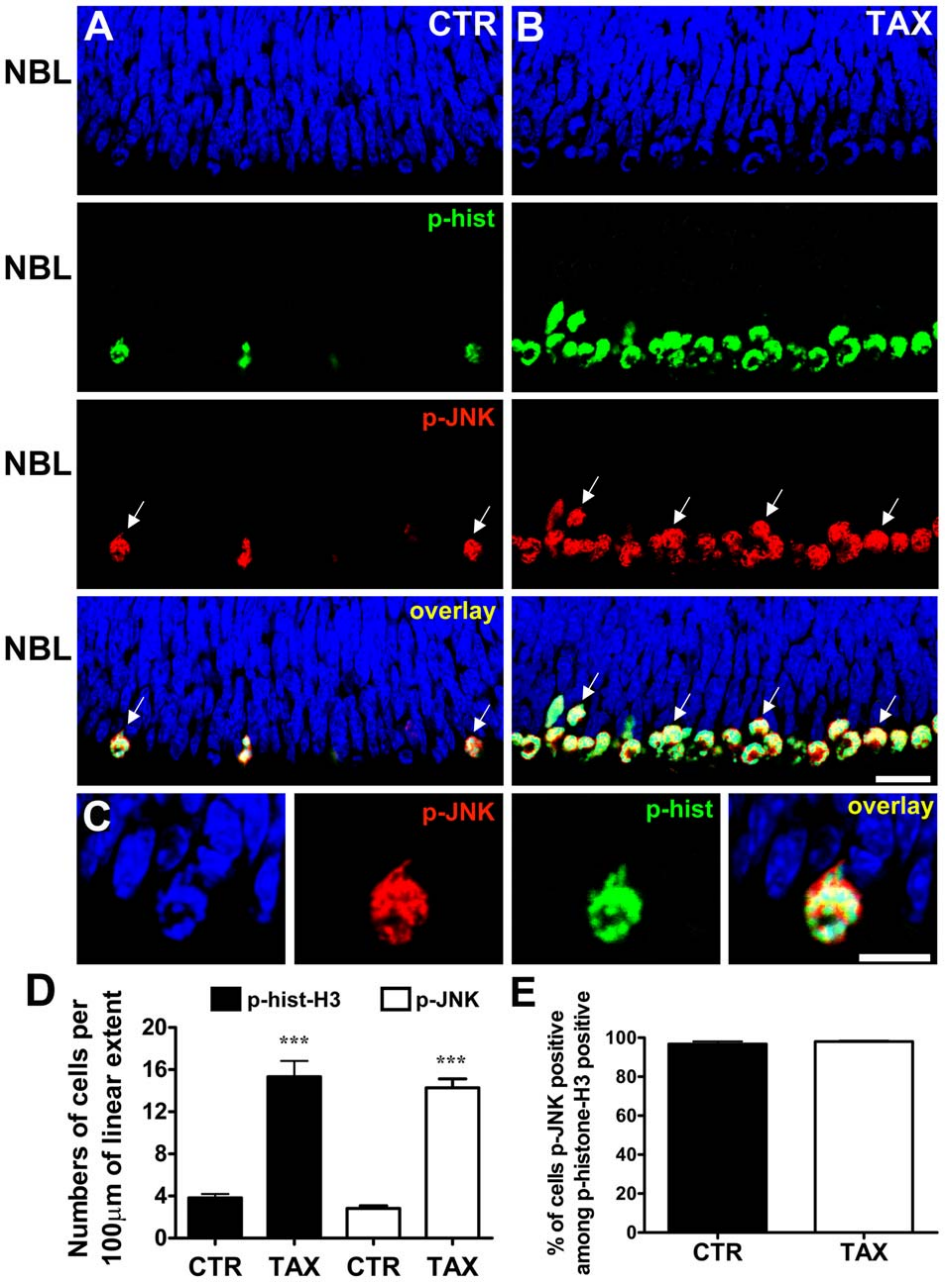
JNK is phosphorylated during mitosis of retinal progenitor cells. (A, B) Representative confocal photomicrographs of immunohistochemistry for phospho-JNK (red) and phospho-histone-H3 (green) in sections of retinal tissue maintained for 3 hours *in vitro* either in absence (A - CTR) or presence of taxol (B - TAX) showing the NBL. The sections were counterstained with DAPI (blue). Arrows indicate examples of cells double stained for phospho-JNK and phospho-histone-H3. Scale bar: 20 µm. (C) Higher magnification of mitotic cell showing the subcellular localization of phospho-JNK (red), phospho-histone-H3 (green), and DAPI (blue). Scale bar: 10 µm. (D) Numbers of phospho-JNK or phospho-histone-H3 stained cells per 100 µm of linear extent parallel to the retinal surface, along the mitotic stratum of retinal explants maintained *in vitro* for 3 hours, either in the absence (CTR) or presence of taxol (TAX). (E) Percentage of cells stained for phospho-JNK among all cells immunolabeled for phospho-histone-H3 in the mitotic stratum. Data are means±S.E.M. from three independent experiments. CTR - control; TAX - taxol; NBL - neuroblastic layer; *** *P*<0.001 versus CTR.

We first examined the phosphorylation of JNK in retinal explants of newborn rats (postnatal day 1 – P1) maintained *in vitro* for 3 hours, and double stained with the phospho-JNK and phospho-histone-H3 antibodies. Immunohistochemical analysis of retinal sections showed that cells in the outer margin of the retinal tissue, where cells undergo mitosis, are double stained with antibodies against both phosphorylated proteins (fig. **1 A**). This suggests that JNK is phosphorylated during mitosis of retinal progenitor cells. In addition, phospho-JNK staining was also detected in the GCL (data not shown).

We then examined the subcellular localization of phosphorylated JNK. Partial overlap was detected between phospho-JNK and either the DNA marker or phospho-histone-H3 staining (fig. **1 C**). These data suggest that JNK is activated in the cell body during mitosis of progenitor cells in developing retina.

Because JNK phosphorylation can be induced by cellular stress, we examined the phosphorylation of JNK in freshly fixed retinal tissue from rats at embryonic day 14 (E14), 18 (E18), 21 (E21) and at postnatal day 1 (P1), by double staining with the phospho-JNK and phospho-histone-H3 antibodies. Cells in the outer margin of the retinal tissue were double stained with antibodies against both phosphorylated proteins only at E21 and P1 (fig. **S1 C** and **D**). The staining at E21 was lighter than at P1. In addition, little overlap was detected between phospho-JNK and either the DNA marker or phospho-histone-H3 staining at E21 (fig. **S1 C**). These data suggest that JNK is activated in the cellular process during mitosis of progenitor cells in developing retina at E21. We found no cell stained for phospho-JNK in the mitotic stratum in the outer margin of the neuroblastic layer, nor were there any cells double stained for phospho-JNK and phospho-histone-H3 in rats at E14 and E18 (fig. **S1 A** and **B**). Staining was detected in the GCL at all ages analyzed (data not shown). In addition, we examined whether JNK is phosphorylated in the rat cerebral cortex, but no labeled cells were found in sections from P7 rat brains (data not shown). These data suggest that JNK is phosphorylated during mitosis of progenitor cells in the retina of rat only at E21 and P1.

To confirm that JNK is phosphorylated during mitosis, retinal explants were maintained *in vitro* for 3 hours in the presence of taxol that induces an arrest of the cell cycle in mitosis due to inhibition of microtubule depolymerization [50]. Treatment with taxol led to an increase in the number of phospho-JNK positive cells in the mitotic stratum, from a mean±S.E.M. of 2.83±0.25 to 14.28±0.85 cells per 100 µm of linear extension along the outer margin of the retina (fig. **1 B** and **D**). Similarly, the number of phospho-histone-H3 positive cells increased from 3.82±0.36 to 15.32±1.50 cells per 100 µm, as expected (fig. **1 B** and **D**). Similar results were seen after staining with an antibody for total JNK (data not shown). In control conditions, 96.8%±1.3 of the cells stained for phospho-histone-H3 were also stained for phospho-JNK, and a similar proportion (98.0%±0.4) was observed in the retina treated with taxol (fig. **1 E**). These results confirm that JNK is phosphorylated during mitosis of retinal progenitor cells in the newborn rat.

### JNK1 and/or JNK2 are preferentially phosphorylated during mitosis of retinal progenitor cells

**Figure 2:**
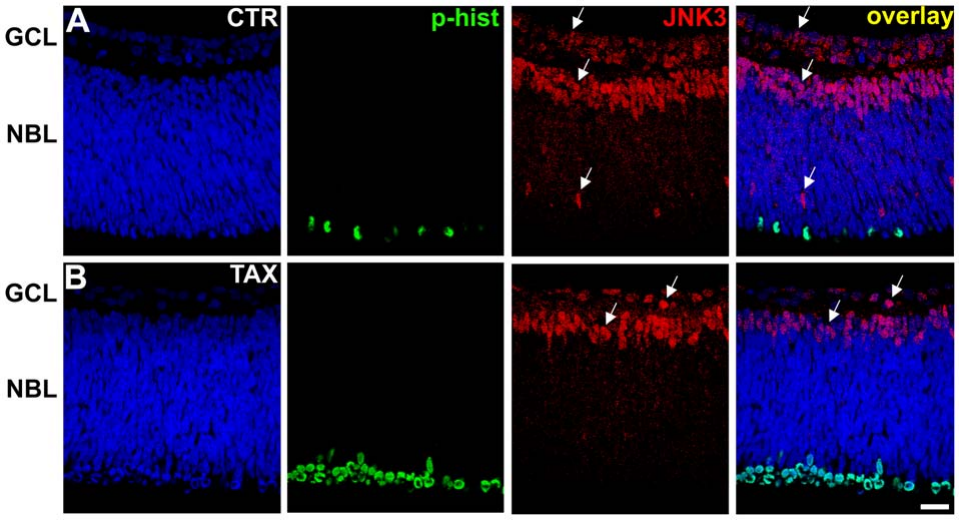
JNK3 is absent during mitosis of retinal progenitor cells. Representative confocal photomicrographs of immunohistochemistry for JNK3 (red) and phospho-histone-H3 (green) in sections of retinal tissue maintained for 3 hours *in vitro* either in absence (A - CTR) or presence of taxol (B - TAX). The sections were counterstained with DAPI (blue). Cells stained for JNK3 were found only in the ganglion cell layer (GCL), in the immature inner nuclear layer and in a few cells in the NBL. Arrows indicate examples of JNK3 positive cells. Scale bar: 20 µm. CTR - control; TAX - taxol; GCL - ganglion cell layer; NBL - neuroblastic layer.

We examined the expression and phosphorylation of the various isoforms of JNK in retinal explants maintained *in vitro* for 3 hours, either in the absence or presence of taxol. Sections of retinal explants were double stained with an antibody that recognizes only the JNK3 isoform and with the antibody to phospho-histone-H3. We found no cell stained for JNK3 in the mitotic stratum in the outer margin of NBL, nor was there any cell double stained for JNK3 and phospho-histone-H3 (fig. **2 A** and **B**). JNK3 positive cells were found only in the GCL, in the immature inner nuclear layer and in scattered profiles within the NBL, possibly horizontal cells, as shown by DAPI staining (fig. **2 A** and **B**). Treatment with taxol did not alter JNK3 staining (fig. **2 B**), whereas the number of cells stained for phospho-histone-H3 increased (fig. **2 B**), as expected. These data suggest that JNK3 is not expressed during mitosis of retinal progenitor cells.

**Figure 3:**
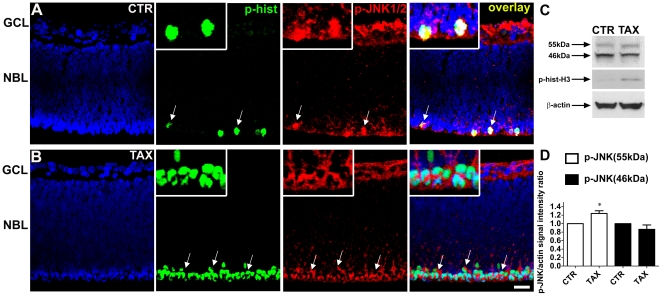
JNK1 and/or JNK2 are preferentially phosphorylated during mitosis of progenitor cells in developing retina. (A, B) Representative confocal photomicrographs of immunohistochemistry for phospho-JNK1/2 (red) and phospho-histone-H3 (green) in sections of retinal tissue maintained for 3 hours *in vitro* either in absence (A - CTR) or presence of taxol (B - TAX). The sections were counterstained with DAPI (blue). Cells double stained for phospho-JNK1/2 and phospho-histone-H3 are indicated with arrows. Scale bar: 20 µm. Inset: higher magnification of mitotic cells. (C) Representative western blot of phosphorylated JNK in total protein extracts of retinal explants maintained for 3 hours *in vitro* either in absence (CTR) or presence of taxol (TAX). The upper blot was done with the antibody for phospho-JNK, the middle blot with an antibody for phospho-histone-H3, and the lower blot with an antibody for β-actin as loading control. (D) Densitometric analysis of the western blot of phospho-JNK. Data are means±S.E.M. from three independent experiments. CTR - control; TAX - taxol; GCL - ganglion cell layer; NBL - neuroblastic layer. * *P*<0.05 versus CTR.

We next used an antibody that recognizes both phosphorylated isoforms JNK1 and JNK2. We found cells double stained for phospho-JNK1/2 and phospho-histone-H3 in the mitotic stratum (fig. **3 A**). In addition, staining was also detected in the GCL (fig. **3 A**). The pattern of staining for phospho-JNK1/2 (fig. **3 A**) was similar to the staining with the antibody that recognizes all three phosphorylated isoforms of JNK (fig. **1 A**). Treatment with taxol increased the number of cells double stained for phospho-JNK1/2 and phospho-histone-H3 in the mitotic stratum, but did not alter the staining for phospho-JNK1/2 in other cells (fig. **3 B**). Although treatment with taxol changed the subcellular localization of phospho-JNK1/2 (fig. **3 B**), it did not change the pattern of staining with the antibody that recognizes all three phosphorylated isoforms of JNK (fig. **1 B**). Thus, we could not confirm the change in subcellular localization of JNK. However, it is clear that treatment with taxol led to an increase in the number of mitotic cells stained for phospho-JNK1/2. These data suggest that JNK1 and/or JNK2 are phosphorylated during mitosis of retinal progenitor cells.

In an attempt to differentiate which isoform is phosphorylated during mitosis, we examined western blots of phospho-JNK from total protein extracts of retinal explants maintained *in vitro* for 3 hours. Western blot allows the distinction between the isoforms JNK1 (46 kDa) and JNK2 (55 kDa) [9], [11], [51]. Treatment with taxol increased by approximately 24% the amount of phosphorylated JNK2, but did not alter the amount of phosphorylated JNK1 (fig. **3 C** and **D**). Since phosphorylated JNK1 and/or JNK2 were increased only in mitotic cells (fig. **3 B**), and there was no apparent expression of JNK3 nor changes in the phosphorylation of JNK in other cells, the change in phosphorylation of JNK in western blots that follows treatment with taxol is most likely restricted to the mitotic cells. As a control that taxol arrested the proliferating cells in mitosis, treatment with taxol increased the phosphorylation of histone-H3, as expected (fig. **3 C**). These data suggest that isoform JNK2 could be preferentially phosphorylated during mitosis of progenitor cells in developing retina.

### JNK is preferentially phosphorylated during early stages of mitosis in retinal progenitor cells

**Figure 4:**
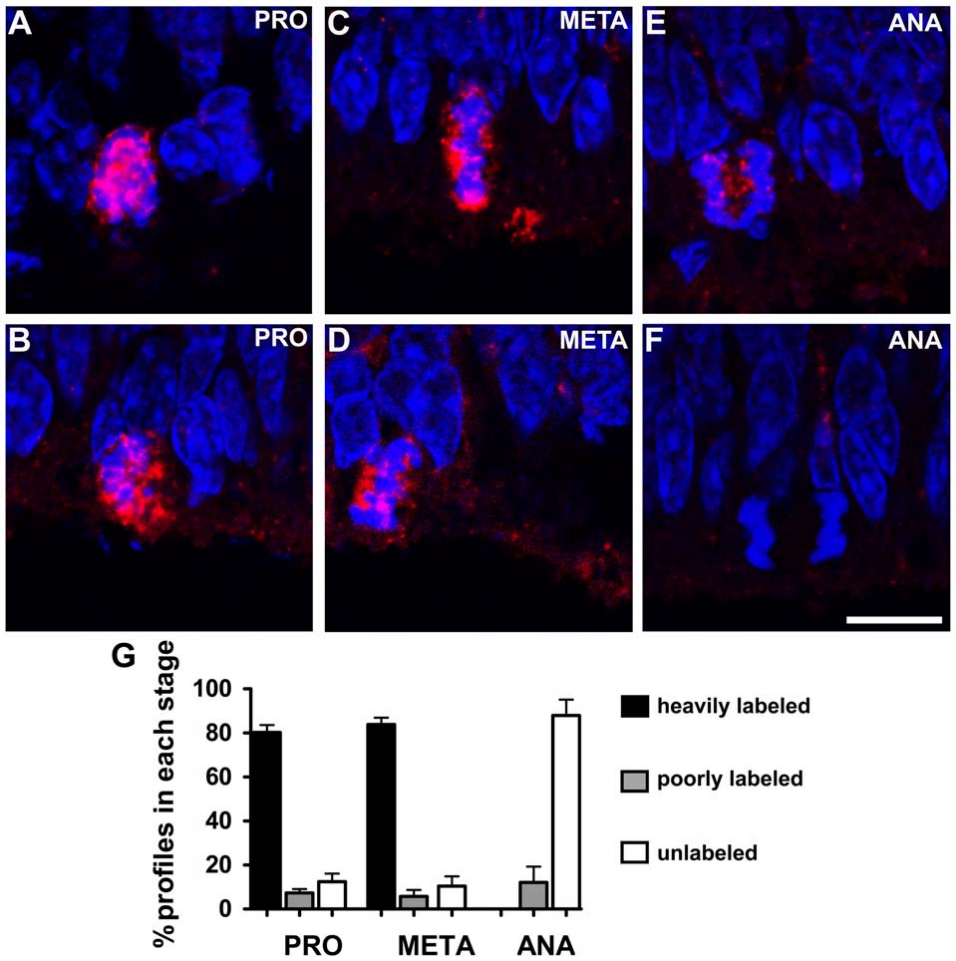
JNK is phosphorylated preferentially during early stages of mitosis in retinal progenitor cells. (A–F) Representative confocal photomicrographs of immunolabeling for phospho-JNK (red) in sections of retinal tissue maintained 3 hours *in vitro* showing mitotic cells. The sections were counterstained with DAPI (blue). (A, B) Cells in prophase/prometaphase heavily labeled for phospho-JNK. (C, D) Cells in metaphase heavily labeled for phospho-JNK. (E, F) Cells in anaphase either poorly labeled or unlabeled for phospho-JNK. Scale bar: 10 µm (G) Quantification of phospho-JNK staining in different mitotic stages. Mitotic stages were classified according with chromatin morphology in prophase/prometaphase (PRO), metaphase (META) or anaphase (ANA) and phospho-JNK staining were classified in heavily labeled, poorly labeled and unlabeled. The graph represents pools of at least 250 mitotic cells in each experiment. Data are means±S.E.M. from three independent experiments.

Microscopic examination of chromatin morphology with the use of the DNA marker DAPI helps to distinguish among stages of the M-phase: in prophase/prometaphase, chromosomes are condensed and spread throughout the cell body; in metaphase, the condensed chromosomes are clearly aligned at the metaphase plate; and in anaphase, symmetrically diverging chromosomes are easily detected. We examined the phosphorylation of JNK in distinct mitotic stages in sections of retinal explants double stained with an antibody against to phospho-JNK and counterstained with DAPI. Approximately 80% of the prophase/prometaphase plus metaphase profiles were heavily stained for phospho-JNK (fig. **4 A–D** and **G**). In contrast, almost all cells in anaphase were either unlabeled (fig. **4 F** and **G**) or poorly labeled (fig. **4 E** and **G**). These data showed that JNK is preferentially phosphorylated during early stages of mitosis in retinal progenitor cells.

### Inhibition of JNK induces mitotic arrest

**Figure 5:**
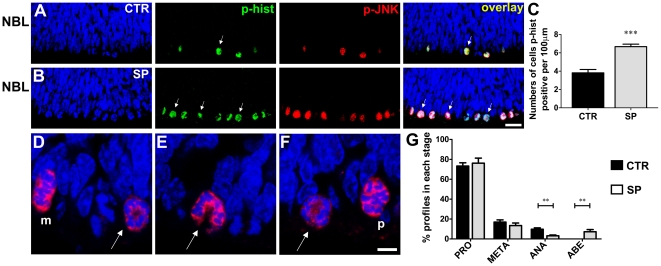
Inhibition of JNK induces mitotic arrest. (A, B) Representative confocal photomicrographs of immunohistochemistry for phospho-JNK (red) and phospho-histone-H3 (green) in sections of retinal tissue maintained for 3 hours *in vitro* either in absence (A - CTR) or presence of SP600125 (B - SP). The sections were counterstained with DAPI (blue). Arrows indicate examples of phospho-histone-H3 positive cells. Scale bar: 20 µm. (C) Numbers of phospho-histone-H3 stained cells per 100 µm of linear extent parallel to the retinal surface, along the mitotic stratum of retinal explants maintained *in vitro* for 3 hours either in the absence (CTR - black column) or presence of SP600125 (SP - gray column). *** *P*<0.001 versus CTR. (D–F) Representative confocal photomicrographs of mitotic cells immunolabeled for phospho-JNK (red) in sections of retinal tissue maintained for 3 hours *in vitro* in the presence of SP600125. The sections were counterstained with DAPI (blue). Stages of mitosis are indicated as either prophase/prometaphase (p) or metaphase (m), while arrows show aberrant chromosome morphology. Compare the disorganized chromatin with the profiles shown in fig. 4. Scale bar: 5 µm. (G) Frequency of prophase/prometaphases (PRO), metaphases (META), anaphases (ANA) and aberrant profiles (ABE) among all mitotic cells in the outer margin of retinal explants maintained *in vitro* for 3 hours either in the absence (CTR - black columns) or presence of SP600125 (SP - gray columns). Data are means±S.E.M. pooled from at least 250 mitotic cells in each of six independent experiments. CTR - control; SP - SP600125; NBL - neuroblastic layer. ** *P*<0.01 versus CTR.

The phosphorylation of JNK in early stages of the M-phase suggested that activation of JNK might be important to mitotic progression in retinal progenitor cells. To test this hypothesis, we maintained retinal explants *in vitro* for 3 hours either in the absence or presence of SP600125 (20 µM), an inhibitor of JNK activity, which does not prevent the phosphorylation of the enzyme [52]. The inhibitor of JNK induced an accumulation of phospho-histone-H3 positive cells in the mitotic stratum, from 3.82±0.36 to 6.67±0.27 cells per 100 µm of linear extension along the outer margin of the retina (fig. **5 A**–**C**). Similarly, there was an accumulation of phospho-JNK positive cells in the mitotic stratum from 2.83±0.25 to 6.73±0.79 cells (fig. **5 A** and **B**). Almost all cells positive for phospho-histone-H3 were also positive for phospho-JNK (fig. **5 A** and **B**). These data suggest that inhibition of JNK induces an accumulation of mitotic cells in the mitotic stratum in developing retinal tissue.

We next examined chromosome morphology in the mitotic cells. Following treatment with the JNK inhibitor, condensed chromosomes seemed less orderly organized than in control retinal tissue, and were often displaced to one side of the cell. In several cases, the chromosomes were arranged in a doughnut-like shape. All such profiles were scored as aberrant (fig. **5 D**–**F** - arrows). Nevertheless, in most cases, chromosomes in the prophase/prometaphase and metaphase could still be clearly distinguished (fig. **5 D**, **F** and **G**). In most cells with aberrant chromosome morphology, the centrosome, labeled with an antibody for γ-tubulin protein [53], was located at the center of the chromatin instead of the pole of cell (fig. **S2**). This abnormal localization of the centrosome may explain the aberrant chromosome morphology seen in mitotic cells after treatment with the JNK inhibitor.

Inhibition of JNK led to a decrease in the number of anaphase profiles (fig. **5 G**), without any apparent change in the frequency of prophase/prometaphases and metaphases (fig. **5 G**). These data suggest that the mitotic arrest induced by JNK inhibition occurs either prior to, or at the metaphase-anaphase transition.

## Discussion

This study addressed the behavior of JNK during the M-phase of the cell cycle of retinal progenitor cells. The results showed that: (a) In the retina of newborn rats (P1), JNK is phosphorylated mainly during the early stages of mitosis; (b) The major isoforms involved in this effect are JNK1 and/or JNK2. (c) Inhibition of JNK induced cell cycle arrest in mitosis. (d) Treatment with the JNK inhibitor decreased the number of cells in anaphase, but did not alter the number of cells in either prophase/prometaphase or metaphase. (e) Cells with aberrant chromatin morphology were found after treatment with the JNK inhibitor.

Cells in the outer margin of retinal tissue, where mitosis occurs, were double labeled for phospho-JNK and phospho-histone-H3, and showed condensed chromatin typical of M-phase. The phosphorylation of JNK in mitotic profiles was observed both in retinas cultured *in vitro* and in freshly fixed retina *(in situ)*, showing that labeling in the explants was not due to the separation of the retina from the pigment epithelium. We detected JNK phosphorylation during mitosis of progenitor cells in the retina *in situ* at E21 and P1. The fact that JNK phosphorylation occurs during mitosis only after E21 and that phospho-JNK staining was lighter at E21 when compared with P1, suggests that JNK phosphorylation may be temporally regulated during retina development. We could not detect JNK phosphorylation in M-phase of neural progenitor cells in the cerebral cortex from P7 rats. This result suggests that JNK activation during mitosis may not be a universal event and may be unique in the retina. However, as the JNK phosphorylation appears to be regulated during retinal development, similar mechanisms may occur in the cerebral cortex. Therefore, a more detailed analysis will be necessary to describe the occurrence of phosphorylation of JNK in other areas of the CNS.

Treatment with taxol, that induces cell cycle arrest in mitosis, increased the number of cells double labeled for phospho-JNK and phospho-histone-H3. These data strongly suggest that JNK is phosphorylated during mitosis of retinal progenitor cells. Our immunohistochemical analysis points to JNK1 and/or JNK2 as preferentially phosphorylated among the 3 isoforms of JNK in M-phase of retinal progenitors. Western blot suggested that, after taxol treatment, there is an increase in the phosphorylation status only in the JNK2 isoforms. The small increase of only approximately 24%, may be due to the fact that cells in M-phase are only a small fraction of the retinal tissue and a consistent increase of JNK phosphorylation with taxol was restricted to M-phase cells, among a lighter staining of other cells. Thus the results of the western blot may have been masked by phospho-JNK staining in other cells. Our data suggest that JNK2 may be preferentially activated during mitosis of retinal progenitor cells, and therefore corroborate studies showing that JNK2 is involved with cellular proliferation [20]–[24].

In our study, JNK were preferentially activated in early mitotic stages. Recently, it was reported that JNK activity increases in various cell lines during late G2 phase, and remains high at the onset of mitosis, while in the final stages of mitosis JNK is degraded via proteasome, mediated by the E3 ubiquitin ligase APC/C [54], which is involved in the degradation of key proteins of the cell cycle. Further experiments are needed to test whether APC/C is also involved in the turnover of JNK in the developing retinal tissue, wherein MAP kinase cascades are heavily dependent on cell interactions [55].

Most studies of JNK activation in retinal tissue were done in the context of neurodegeneration [56]–[60]. Our group has previously shown that p38 kinase is also phosphorylated in M-phase of retinal progenitor cells [49]. The current data add further evidence that MAPKs are involved with cellular proliferation in developing retinal tissue, in particular during M-phase. We found that an inhibitor of JNK induced cell cycle arrest at the metaphase/anaphase transition, accompanied by chromatin abnormalities in the retina. In tumor cells, inhibition of JNK, either using a pharmacological inhibitor or deletion of JNK1/2, induced cell cycle arrest in M-phase and polyploidy [37], [40]. Our group has previously shown that inhibition of p38 kinase also increases the number of M-phase cells in the mitotic stratum of retinal tissue and causes chromatin abnormalities [49]. We found no evidence of synergism between both inhibitors of JNK and p38 (data not shown). Therefore, both kinases may work in a partially redundant fashion to promote M-phase progression.

Among downstream targets of JNK, previous work has identified Cdh1, Bcl-2, cytoskeletal proteins, histone-H3, Cdc25 and the transcription factor Sp1 during M-phase [54], [61]–[66]. The relevant substrates and the mechanisms of control of M-phase progression by JNK in the retinal tissue remain, however, to be determined. The abnormal localization of the centrosomal protein γ-tubulin, detected in most cells with aberrant chromosome morphology, may be important for the M-phase arrest of the proliferating cells.

It has been reported that inhibition of JNK2 causes defects in central spindle formation and chromosome segregation during anaphase in mammalian cells in culture [41]. Our data suggest that JNK may be required for the separation or localization of the centrosome in the cell poles, therefore controlling the separation of chromatids at the outset of anaphase, and possibly helping to prevent polyploidy. It has, indeed, been shown that inhibition of JNK results in polyploidy and apoptosis of cultured cell lines [37], [40]. Thus, JNK may be important to maintain genomic stability and prevent programmed cell death in progenitor cells during retinal development. In conclusion, the current study suggests a new role of JNK in the control of progenitor cell proliferation, especially in mitosis, within retinal tissue.

## Supporting Information

Figure S1 **JNK is phosphorylated during mitosis of retinal progenitor cells** ***in situ*** **.** Representative confocal photomicrographs of immunohistochemistry for phospho-JNK (red) and phospho-histone-H3 (green) in sections of freshly fixed retinal tissue from rats at embryonic day 14 (A - E14), 18 (B - E18), 21 (C - E21) and at postnatal day 1 (D - P1) showing the NBL. The sections were counterstained with DAPI (blue). Arrows indicate examples of cells double stained for phospho-JNK and phospho-histone-H3. NBL - neuroblastic layer. Scale bar: 20 µm.
(TIF)

Figure S2 **Aberrant centrossomal localization in mitotic cells following JNK inhibition.** Representative confocal 3D reconstruction photomicrographs of double staining for phospho-JNK (red) and γ-tubulin (green) in sections of retinal tissue maintained *in vitro* for 3 hours in the presence of inhibitor of JNK, showing mitotic cell. In cells with aberrant chromosome morphology the centrosome is located in the center of the chromatin instead of the pole of cell. Scale bar: 5 µm.
(TIF)
