# Supplementary figures and images for: Activation of c-Jun N-Terminal Kinase (JNK) during Mitosis in Retinal Progenitor Cells
Source: PLoS One. 2012 Apr 4;7(4):e34483. doi: 10.1371/journal.pone.0034483 (PMC3319587; doi:10.1371/journal.pone.0034483)

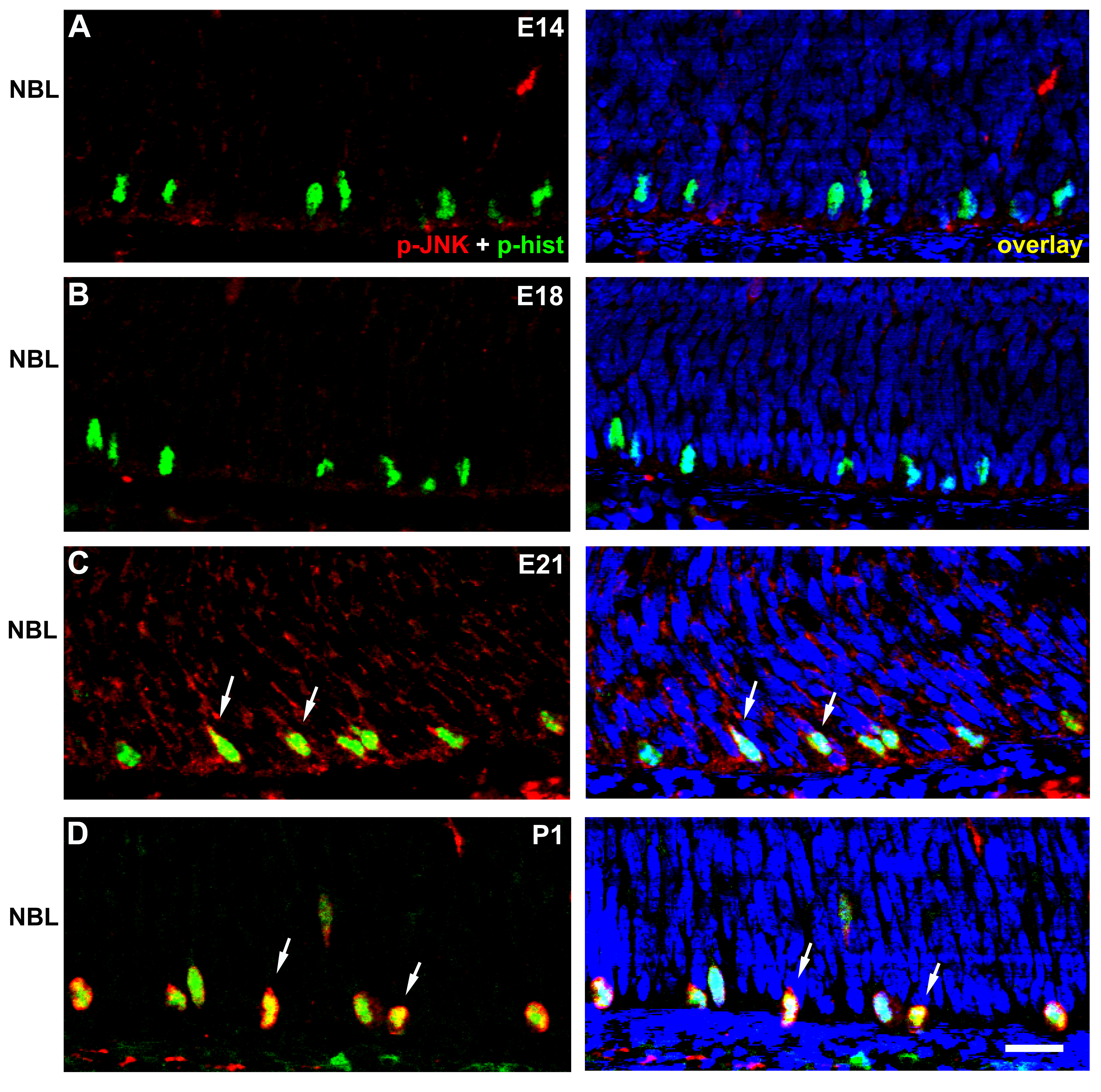

Supplement: Figure S1 — JNK is phosphorylated during mitosis of retinal progenitor cells in situ . Representative confocal photomicrographs of immunohistochemistry for phospho-JNK (red) and phospho-histone-H3 (green) in sections of freshly fixed retinal tissue from rats at embryonic day 14 (A - E14), 18 (B - E18), 21 (C - E21) and at postnatal day 1 (D - P1) showing the NBL. The sections were counterstained with DAPI (blue). Arrows indicate examples of cells double stained for phospho-JNK and phospho-histone-H3. NBL - neuroblastic layer. Scale bar: 20 µm. (TIF) [file pone.0034483.s001.tif]

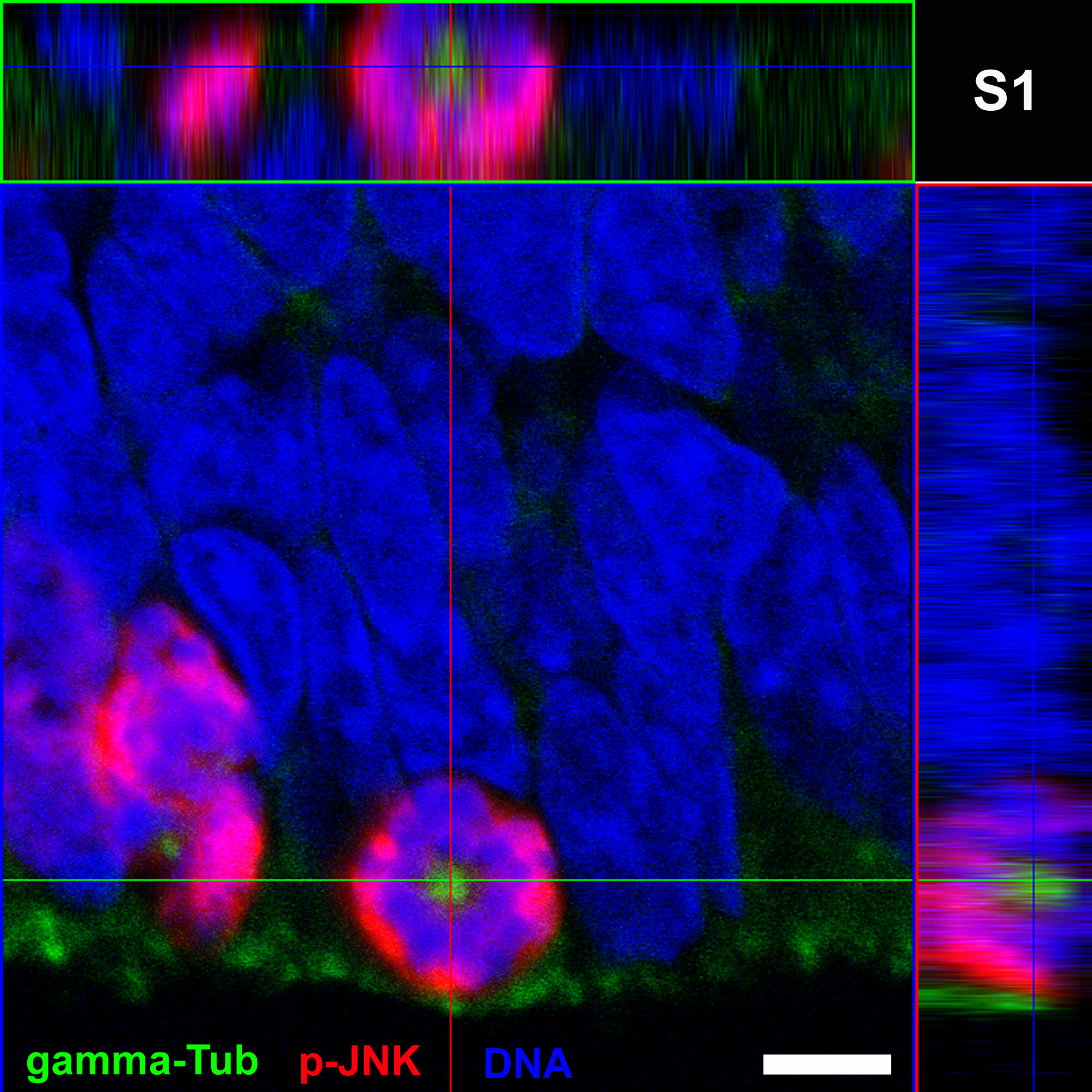

Supplement: Figure S2 — Aberrant centrossomal localization in mitotic cells following JNK inhibition. Representative confocal 3D reconstruction photomicrographs of double staining for phospho-JNK (red) and γ-tubulin (green) in sections of retinal tissue maintained in vitro for 3 hours in the presence of inhibitor of JNK, showing mitotic cell. In cells with aberrant chromosome morphology the centrosome is located in the center of the chromatin instead of the pole of cell. Scale bar: 5 µm. (TIF) [file pone.0034483.s002.tif]
